# Preparation and Performance of Ferric-Rich Bauxite-Tailing-Based Thermal Storage Ceramics

**DOI:** 10.3390/ma16216900

**Published:** 2023-10-27

**Authors:** Qi Wang, Minghao Fang, Xin Min, Pengpeng Du, Zhaohui Huang, Yangai Liu, Xiaowen Wu, Yulin Liu, Changmiao Liu, Feihui Huang

**Affiliations:** 1 Engineering Research Center of Ministry of Education for Geological Carbon Storage and Low Carbon Utilization of Resources, Beijing Key Laboratory of Materials Utilization of Nonmetallic Minerals and Solid Wastes, National Laboratory of Mineral Materials, School of Materials Science and Technology, China University of Geosciences, Beijing 100083, China; wangqi-cugb@foxmail.com (Q.W.); minx@cugb.edu.cn (X.M.); dupengpengai@163.com (P.D.); huang118@cugb.edu.cn (Z.H.); liuyang@cugb.edu.cn (Y.L.); xwwu@cugb.edu.cn (X.W.); 2 Zhengzhou Institute of Multipurpose Utilization of Mineral Resources, Chinese Academy of Geological Sciences, Zhengzhou 450006, China; lantns@163.com (Y.L.); changmiaoliu@163.com (C.L.); 3 Shandong Aofu Environmental Technology Co., Ltd., Dezhou 251599, China; huangfeihui@hotmail.com

**Keywords:** bauxite tailings, thermal storage ceramic, specific heat capacity, bulk density, RTO

## Abstract

In recent years, regenerative thermal oxidizer (RTO) has been widely used in the petroleum industry, chemical industry, etc. The massive storage required by solid waste has become a serious problem. Due to their chemical composition, bauxite tailings as raw materials for high-temperature thermal storage ceramics show enormous potential in the fields of research and application. In this study, we propose a method for preparing ferric-rich and high specific storage capacity by adding Fe_2_O_3_ powder to bauxite tailings. Based on a 7:3 mass ratio of bauxite tailings to lepidolite, Fe_2_O_3_ powder with different mass fractions (7 wt%, 15 wt%, 20 wt%, 30 wt%, and 40 wt%) was added to the ceramic material to improve the physical properties and thermal storage capacity of thermal storage ceramics. The results showed that ferric-rich thermal storage ceramics with optimal performance were obtained by holding them at a sintering temperature of 1000 °C for 2 h. When the Fe_2_O_3_ content was 15 wt%, the bulk density of the thermal storage ceramic reached 2.53 g/cm^3^, the compressive strength was 120.81 MPa, and the specific heat capacity was 1.06 J/(g·K). This study has practical guidance significance in the preparation of high thermal storage ceramics at low temperatures and low costs.

## 1. Introduction

Bauxite tailings refer to the solid wastes produced in the process of mining and the beneficiation of bauxite. On average, 0.25 t of tailing is produced for every 1 t of bauxite [1,2,3,4]. Due to the large production capacity of industries, complex composition and the structure of tailings, the main disposal method of tailings is storage in tailings dams [5]. In China, there is a huge market and demand for metal aluminum, alumina, and aluminum refractory materials, and the production of bauxite tailings is also up to tens of millions of tons per year [6,7,8,9,10,11,12]. The utilization and consumption of bauxite tailings are of great social and economic significance.

Bauxite tailings are mainly composed of Al_2_O_3_ and SiO_2_, and bauxite tailings from different mining and production areas may also contain Fe_2_O_3_, TiO_2_, and other substances. Bauxite tailings via the beneficiation Bayer process may also contain some Na_2_O [13,14,15,16,17,18]. There are multiple methods to utilize bauxite tailings that possess complex chemical compositions. Zhang et al. [19] extracted aluminum and lithium elements from bauxite tailings using acid leaching, but as a result, new waste was generated. Ou et al. [20] added quicklime to bauxite tailings to provide a reference for the practical application of drainage solidification engineering. Zhou et al. [21] applied it to the preparation of building materials using 3D printed mortar, but the addition of bauxite tailings may deteriorate the material’s performance as well. Qiang et al. [22] obtained zeolite X using hydrothermal synthesis and a certain amount of bauxite tailings, but high equipment costs and technical difficulties were reported. Mendes et al. [23] collaborated on the preparation of clay bricks using bauxite tailings and iron tailings, but there were issues with high preparation temperatures and the low added value of the product. It is particularly important to find bauxite-tailing-based materials that can be prepared at low temperatures and that exhibit high performance.

Regenerative thermal oxidizers (RTOs), common equipment used for treating waste gases containing organic compounds, are widely used in industries such as petroleum and chemical engineering [24,25,26]. Thermal storage ceramics are an important component of RTO. Because of the high specific heat capacity and thermal stability of mullite and corundum, they are the main phase choice of thermal storage ceramics, so the raw materials of thermal storage ceramics are usually bauxite and potassium feldspar or other fluxes. However, thermal storage ceramics, such a mature industrial products, also have their problems in use: first, the use of high-quality raw materials and higher sintering temperature make the ceramic preparation cost high, and second, the low bulk density of thermal storage ceramics makes the heat storage equipment usually huge, and improving the bulk density of thermal storage ceramics can improve the space utilization efficiency of the equipment or the heat storage per unit volume [27]. Fe_2_O_3_, as a high specific gravity component and a common component in solid waste, has entered the field of researchers. Bauxite tailing is a low-cost material with high Fe_2_O_3_ content, and its characteristics of transforming to mullite and corundum have great potential in improving the above shortcomings of thermal storage ceramics. Fe_2_O_3_ can also improve the bulk density of ceramics to solve the problem of low heat storage per unit body of ceramics. This article uses bauxite tailings as the main raw material and lepidolite as the fluxing agent to explore the effects of different Fe_2_O_3_ additions on the physical properties, preparation, and usage performance of ferric-rich bauxite-tailing-based ceramics. Finally, an excellent and industry-standard ferric-rich bauxite-tailing-based thermal storage ceramic material was prepared.

## 2. Experimental Procedure

### 2.1. Raw Materials

The bauxite tailings were provided by a mining area in Henan Province, China, and lepidolite was obtained from a mining area in Shandong Province. The composition of the two raw materials was analyzed and tested using XRF and the content of Li was obtained using ICP, as shown in Table 1. Fe_2_O_3_ powder with a purity of 99.99% was purchased from Aladdin Company (Jebel Ali, United Arab Emirates).

Table 2 shows the ceramic material’s ingredients. The maximum content of Fe_2_O_3_ is set up at 40 wt%, as excessive Fe_2_O_3_ content increases the cost of ceramic preparation, which is contrary to the original research intention. Moreover, excessive Fe_2_O_3_ content can also lead to low contents of Al_2_O_3_, SiO_2_, and other substances, thereby deteriorating the mechanical properties of ceramics and not meeting the requirements in the actual application environment.

**Figure 1:**
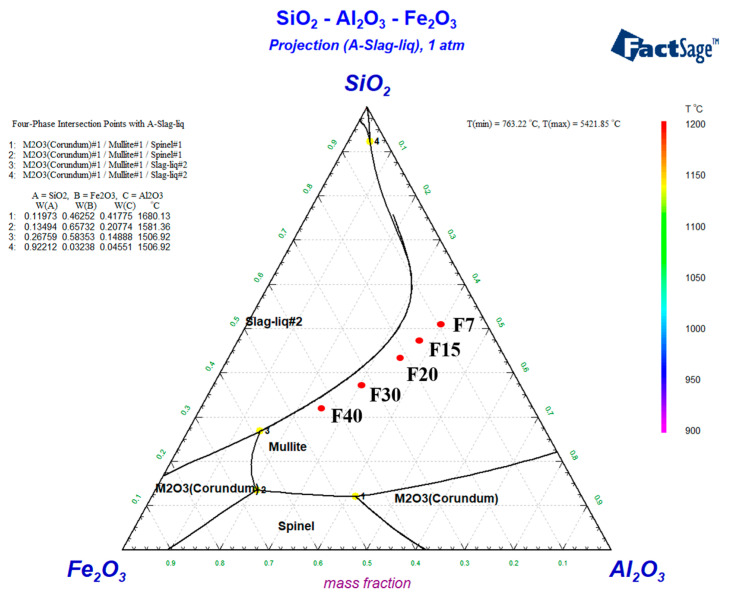
Ternary phase diagram of SiO_2_-Al_2_O_3_-Fe_2_O_3._ This is how the factsage 8.1 calculation results are represented. The #2 in liquid#2 is used to distinguish a single liquid phase Liquid #1 that forms when the temperature is high enough.

The phase diagram of the samples was calculated by factsage 8.1. Because the sum of Al_2_O_3_, SiO_2_ and Fe_2_O_3_ of the samples is about 90 wt%, in order to simplify the calculation, each component is multiplied by 1.3 to simulate. The calculation results of component positions of different samples are shown in Figure 1. The locations of the samples are all in the region of the mullite phase, so the main crystalline phase of the different ceramics is mullite. Mullite can help to maintain high mechanical strength and specific heat capacity properties of ceramics.

Through the horizontal test and comparison of a certain kind of mullite-phase honeycomb heat accumulator ceramic sold, it can be found that its bulk density is 2.41 g/cm^3^, the compressive strength is 103.2 MPa, and the specific heat capacity is 940.2 J/(kg·K). Therefore, the ceramic prepared by this work will use cheaper materials to obtain the same or better performance, and can realize economic and social value.

### 2.2. Preparation

The raw material powder was mixed according to the quantities listed in Table 2. The prepared raw material powder was placed into a ball mill tank, and then deionized water was added for 1 h of ball milling. After milling, the materials were dried in a drying oven at 80 °C. The dried ceramic powder was ground and granulated, and a cylindrical ceramic body with a diameter of 20mm (Φ20) and a height of 15 mm was prepared using dry compression molding where the gauge pressure was 5 MPa. At a heating rate of 10 °C/min, ferric-rich bauxite-tailing-based ceramic materials were obtained by holding them at temperatures of 900 °C, 950 °C, 1000 °C, and 1050 °C for 2 h.

### 2.3. Characterization

The chemical compositions of bauxite tailings were measured using an X-ray fluorescence analyzer (XRF, PANalytical Axios, Almelo, The Netherlands). The powder X-ray diffraction (XRD) patterns of the samples were recorded using X-ray powder diffraction (AXS D8 Advance, Bruker, Billerica, MA, USA). Microstructure morphology was observed and studied using field-emission scanning electron microscopy (FESEM, Zeiss supra-55, Oberkochen, Germany). Differential thermal and thermogravimetric analysis of the bauxite tailings were carried out using a synchronous thermal analyzer (STA409PC, Netzsch, Selb, Free State of Bavaria, Germany), and the specific heat capacity of ceramics was conducted by the synchronous thermal analyzer (STA449C, Netzsch, Selb, Free State of Bavaria, Germany). The bulk density, apparent porosity, and water absorption of ceramics were measured using the Archimedes drainage method (Formulas (1)–(3)), and the compressive strength was measured using the Smart Testing machine (WAW-2000E, Koohui, Jinan, China). The linear shrinkage was measured using vernier calipers and calculated (Formula (4)).

(1) $$\rho =\frac{m_{0}}{m_{1}-m_{2}}\rho_{0}$$

(2) $$P=\frac{m_{1}-m_{0}}{m_{1}-m_{2}}\times 100\%$$

(3) $$W=\frac{m_{1}-m_{0}}{m_{0}}\times 100\%$$

(4) $$L=\frac{D_{0}-D_{1}}{D_{0}}\times 100\%$$

*ρ*, *P*, *W*, and *L* represent bulk density, apparent porosity, water absorption and linear shrinkage, respectively. *m*_0_ is the mass of the dry sample, and *m*_1_, *m*_2_ are the mass of the saturated samples in air and in liquid, respectively. *ρ*_0_ is the density of the impregnated liquid at the test temperature. *D*_0_ and *D*_1_ are the diameter of the samples before or after sintering, respectively.

## 3. Results and Discussion

### 3.1. Composition and Phase Analysis of Bauxite Tailings

**Figure 2:**
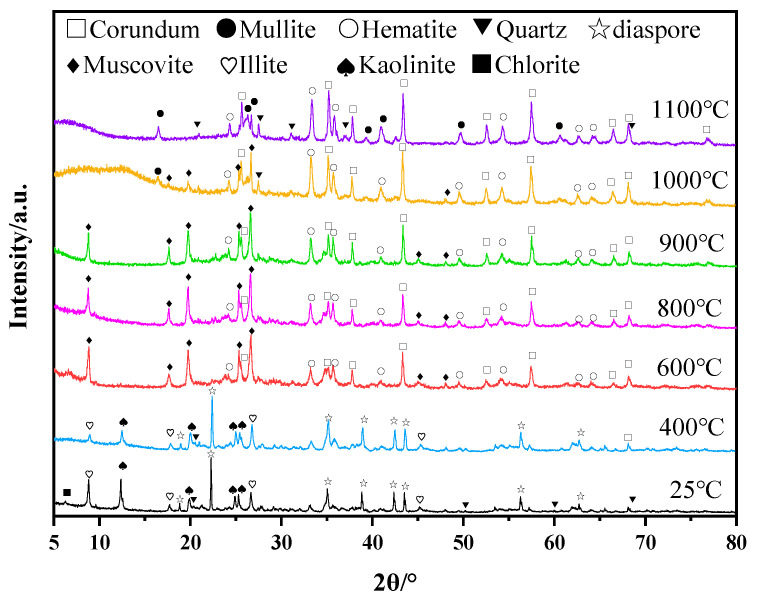
XRD of bauxite tailings and samples after heating treatment at different temperatures.

After the calcination of bauxite tailings at temperatures of 400 °C, 600 °C, 800 °C, 900 °C, 1000 °C, and 1200 °C, the XRD results of the products are shown in Figure 2. At room temperature, the main phases of bauxite tailings are diaspore (Al_2_O_3_·H_2_O), kaolinite (Al_2_O_3_·2SiO_2_·2H_2_O), illite (KAl_2_[(Si,Al)_4_O_10_](OH)_2_), quartz (SiO_2_), and a small amount of hematite (Fe_2_O_3_). After heat treatment at 400 °C, diaspore obtained from bauxite was dehydrated to form free Al_2_O_3_, and kaolinite partially lost structured water to form metakaolinite (Al_2_O_3_·2SiO_2_) [28]. After heat treatment at 600 °C, the free aluminosilicate in the sample generated muscovite according to the obvious diffraction peaks of the muscovite (KAl_2_[(Si,Al)_4_O_10_](OH)_2_) phase that appeared in the XRD. At the same time, the free SiO_2_ oxide formed muscovite. After heat treatment at 1000 °C, the muscovite was transformed into free SiO_2_, and the muscovite transformed into corundum and then reacted with free SiO_2_ to mullite [29]. With the high-temperature vitrification of free SiO_2_ from clays, the liquid phase promoted the formation of mullite (3Al_2_O_3_·2SiO_2_ or 2Al_2_O_3_·SiO_2_). Hematite promoted the formation of the liquid phase in the high-temperature treatment process [30], while hematite crystallized in the process of cooling, resulting in unchanged contents. The main phases in some samples are mullite, corundum, and hematite after heat treatment at 1000 °C and 1100 °C.

**Figure 3:**
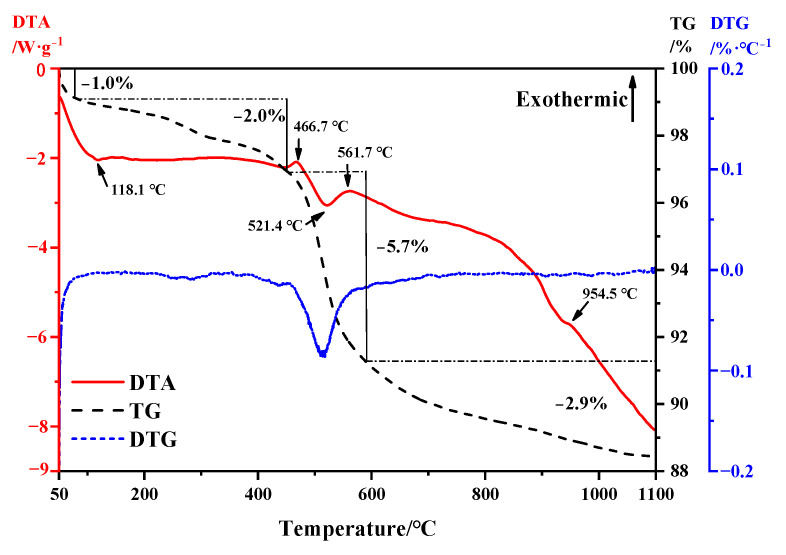
TG-DTA curve of bauxite tailings.

TG-DTA thermodynamic analysis was conducted on bauxite tailings, and the analysis results are shown in Figure 3. From the TG curve, bauxite tailings always exhibit a thermogravimetric reaction at temperatures ranging from room temperature to 1100 °C. It can be observed from the DTG curve that there is obvious thermogravimetric loss between 50 °C and 100 °C, and according to the DTA curve, it indicates that the bauxite tailings are heated and dried and the bound water evaporates at this temperature, and the reaction ends at 118.1 °C. From 118.1 °C to 466.7 °C, the weight loss of bauxite tailings under heat is about 2%. During this period, the kaolinite was partially transformed into metakaolinite. In the temperature range of 466.7–561.7 °C, the sample has a large thermogravimetric phenomenon, the weight loss rate is about 5.7%, and the endothermic valley appears at 521.4 °C. Therefore, the dehydration reaction of diaspore mainly occurs in this temperature range. Combined with Figure 2, it can be observed that muscovite formation reaction also occurred during this period. At 561.7 °C, it is the exothermic peak of muscovite formation reaction. With the increase in temperature, the change of heat and mass is small [31]. At 954.5 °C, there is a small amount of heat release in the sample. The reaction is a combination of decomposition of muscovite and transformation of corundum into mullite.

**Figure 4:**
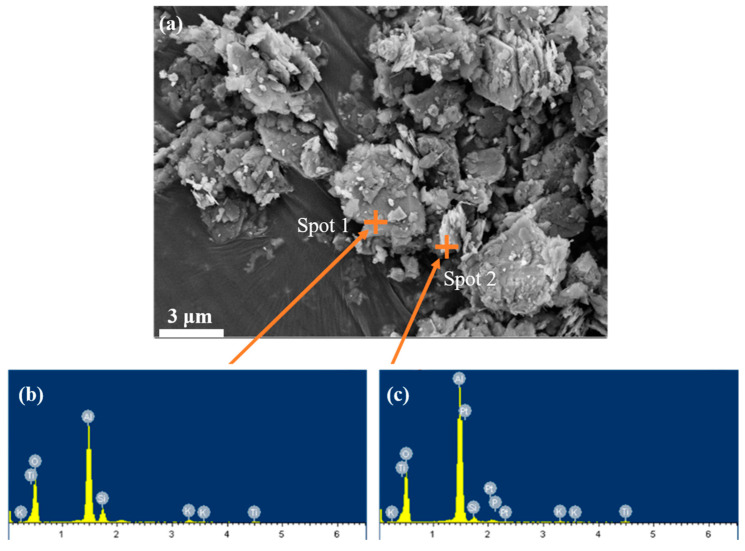
SEM images of bauxite tailings: (**a**) room temperature, (**b**) EDS at spot 1, and (**c**) EDS at spot 2.

The microscopic morphology of bauxite tailing was analyzed by SEM and EDS, and the results were shown in Figure 4. There are different shapes of particles in the figure: some are scaly, some are lumpy. EDS analysis was performed on the scale-like material at spot 2, the main elements of which were O, Al. The bulk material at spot 1 was analyzed by EDS spectrum, and its main elements were O, Al and Si. Due to the accuracy of EDS, it was assumed that the bulk material is quartz, with scaly particles attached to the diaspore or clays such as kaolinite.

**Figure 5:**
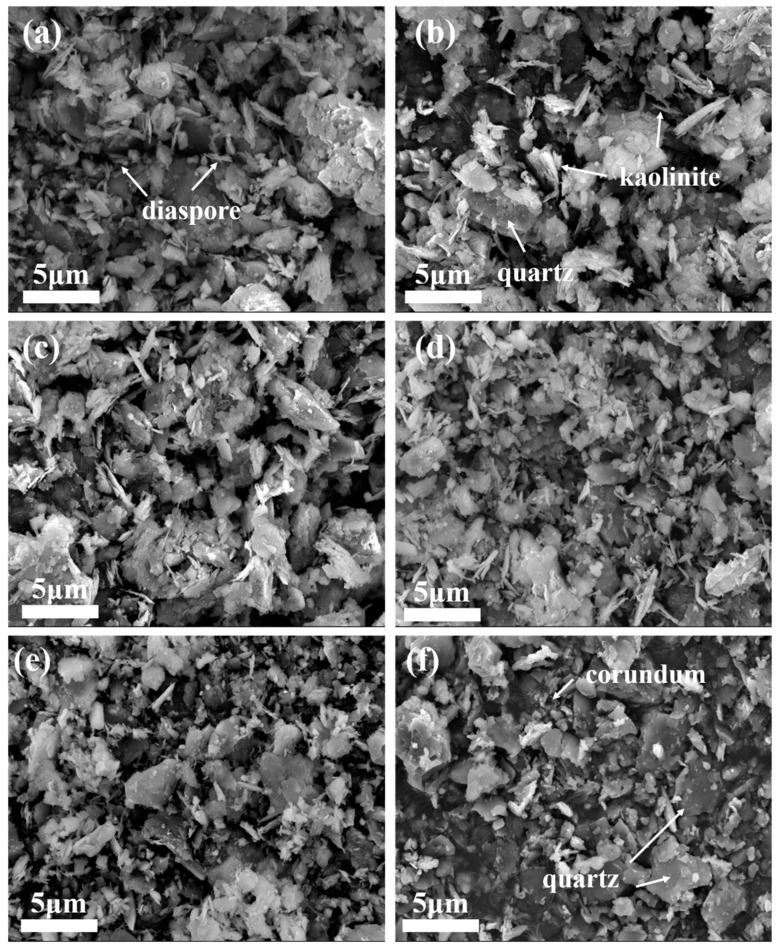
SEM images of bauxite tailings after heating treatment at different temperatures: (**a**) room temperature, (**b**) 600 °C, (**c**) 800 °C, (**d**) 900 °C, (**e**) 1000 °C, and (**f**) 1100 °C.

SEM was applied to observe the morphology of the samples after high-temperature heat treatment, and the results are shown in Figure 5. Figure 5a shows the microstructure of bauxite tailings at room temperature, while Figure 5b–f show the microstructure of bauxite tailings after heating at 600 °C, 800 °C, 900 °C, 1000 °C, and 1100 °C, respectively. Compared with the raw material, it can be observed in the SEM images that the mineral particles in the samples after high-temperature heat treatment remain small, while the number of flake-like particles decreases with an increase in heating temperature. The scale-like particles are mainly diaspore, kaolinite, and other materials, and their decomposition or phase transformation results in a reduction in the displayed phase. As observed in Figure 5f, the aggregation of mineral particles can be observed at a certain degree, indicating that the formation of the liquid phase at this temperature promotes material exchange, phase transformation within the sample, and also the aggregation of particles in the sample, which is conducive to the preparation of ceramics.

### 3.2. Preparation and Performance Study on Ferric-Rich Bauxite-Tailings-Based Thermal Storage Ceramics

**Figure 6:**
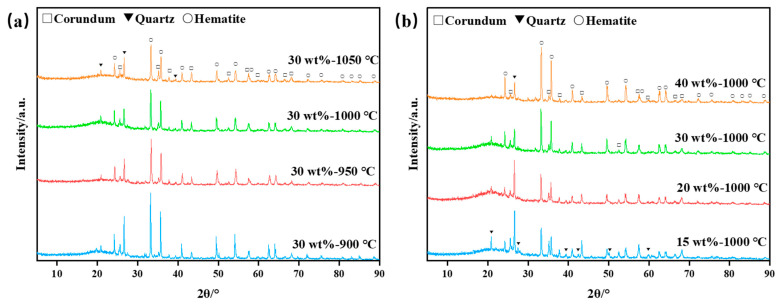
XRD analysis diagrams of bauxite-tailings-based ceramics: (**a**) different sintering temperatures with 30 wt% Fe_2_O_3_ content, and (**b**) different Fe_2_O_3_ contents at sintering temperature of 1000 °C.

XRDs were conducted on bauxite-tailing-based ceramic materials. Figure 6a shows the XRD phase analysis of ceramic materials at different sintering temperatures with a Fe_2_O_3_ content of 30 wt%. It can be observed in the figure that the main phases in the ceramics are corundum, quartz, and hematite after the high-temperature sintering process. With the sintering temperature reaching above 900 °C, the diffraction peak intensities of corundum, quartz, and hematite decrease, indicating that as the temperature increases, increased high-temperature liquid phase production takes place inside the ceramic. With the increase in sintering temperature, the diffraction peak intensity of corundum, quartz, and hematite decreases. It can be inferred that with the increase in sintering temperature, the free SiO_2_ in clays in the ceramic formed a high-temperature liquid phase. The formation of a liquid phase promotes the phase transition efficiency, but at the same time, according to the reduction of crystal volume in Figure 6a, many liquid phases are transformed into amorphous phases. Figure 6b shows the XRD patterns of ceramic materials with different Fe_2_O_3_ contents. As observed in Figure 6b, with an increase in Fe_2_O_3_ content, the intensity of the peaks belonging to hematite increases, while the intensity of the peaks belonging to corundum and quartz decreases. Fe_2_O_3_ content increases, leading to the decreased content of bauxite tailings, indicating that the decrease in the content of corundum and quartz is reasonable. In Figure 6b, the analysis shows that the amorphous phase changes little when the Fe_2_O_3_ concentration is 15–30 wt%. When the concentration is 40 wt%, due to the reduction of clays, the substance that can form high temperature liquid phase is reduced, and the amorphous phase is reduced. Therefore, the addition of Fe_2_O_3_ diluted the concentration of clay minerals, thus the amorphous content dropped. Thus, the relative content of the crystals increased.

**Figure 7:**
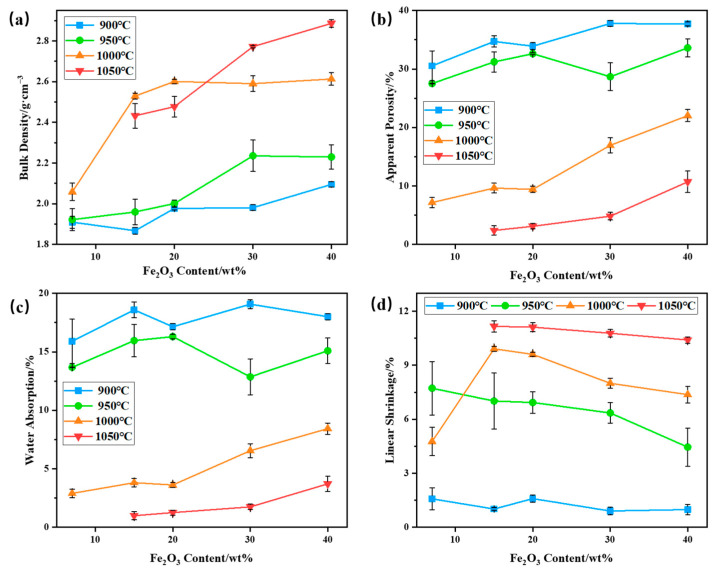
Performances of bauxite-tailings-based ceramic materials with different Fe_2_O_3_ contents at different sintering temperatures: (**a**) volume density, (**b**) apparent porosity, (**c**) water absorption rate, and (**d**) linear shrinkage rate.

The bulk density, apparent porosity, water absorption, and linear shrinkage of bauxite-tailings-based ceramic materials with different Fe_2_O_3_ contents were tested at different sintering temperatures. The results are shown in Figure 7. As shown in Figure 7a, with an increase in Fe_2_O_3_ content, the bulk density of ceramics increases at sintering temperatures of 900 °C, 950 °C, and 1050 °C and initially increases, then it basically remains unchanged at 1000 °C. As sintering temperatures increase, the bulk density first increases and then decreases when Fe_2_O_3_ content is less than 30 wt%. When Fe_2_O_3_ content is 30 wt% and 40 wt%, the bulk density (maximum bulk density = 2.89 g/cm^3^) increases with an increase in sintering temperature. The main reasons for the increase in bulk density are the densification of sintering ceramic and the addition of the Fe_2_O_3_ heavy component. At a sintering temperature of 900 °C, the bulk density and initial shrinkage rate of ceramics are low, while the apparent porosity and water absorption rate are high, indicating that sintering reactions occur less in a ceramic body. When the sintering temperature reaches 950 °C, the bulk density of ceramics increases with an increase in Fe_2_O_3_ content. At this temperature, the linear shrinkage rate of ceramics decreases with Fe_2_O_3_ contents, indicating that the sintering degree decreases with the addition of Fe_2_O_3_. The main reason for the increase in ceramic bulk density is the increase in heavy components. The high levels of apparent porosity and water absorption at 950 °C also indicate a lower sintering degree in ceramics. After sintering the ceramic body at 1000 °C, the bulk density of the ceramic significantly increases with the increase in Fe_2_O_3_, and linear shrinkage first shrinks and then decreases. The apparent porosity and water absorption are lower and increase with an increase in Fe_2_O_3_ content, indicating that 15 wt% and 20 wt% Fe_2_O_3_ can promote the densification of ceramics at this sintering temperature. When the Fe_2_O_3_ content is 15 wt%, the bulk density of the ceramic is 2.53 g/cm^3^, the apparent porosity is 9.64%, the water absorption is 3.81%, and the linear shrinkage is 9.92%. Under this preparation conditions, the bulk density of the ceramic is higher than that of sell thermal storage ceramic products, which is 2.41 g/cm^3^. And to achieve this performance of mullite–corundum composite ceramics, the preparation temperature is usually not less than 1200 °C. When no additional Fe_2_O_3_ was added, due to the fluxing of lepidolite, the liquid phase of the ceramic was too much at 1000 °C, and the macro appearance expands, so the liner shrinkage rate increases first and then decreases with the increase in temperature. When Fe_2_O_3_ was added, the high temperature liquid phase in the ceramic was reduced, and the ceramic expansion is weakened, so the liner shrinkage rate of the wire increases sharply. When the sintering temperature is 1050 °C, the bulk density, apparent porosity, and water absorption of the ceramic increase with an increase in Fe_2_O_3_ content, exhibiting a maximum bulk density of 2.89 g/cm^3^. At this temperature, the linear shrinkage rate of ceramics is relatively high. Therefore, Fe_2_O_3_ has a significant promoting effect on the increase in the bulk density of bauxite-tailings-based ceramics.

**Figure 8:**
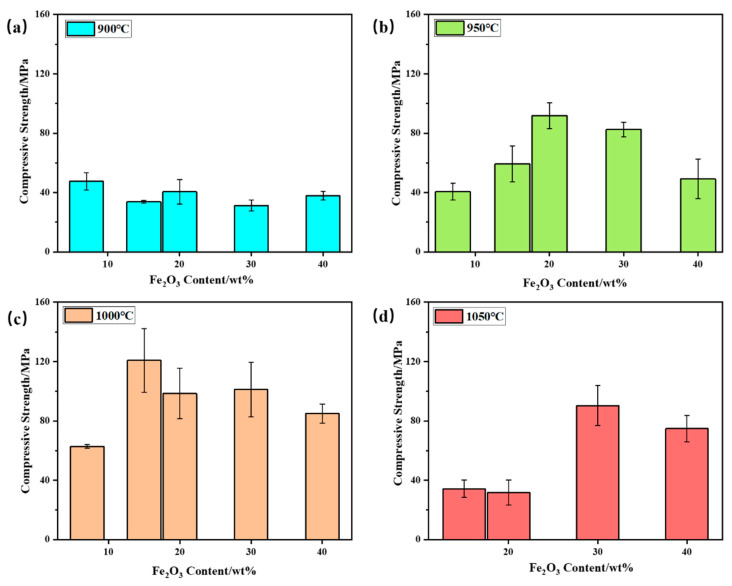
Compressive strength of bauxite-tailings-based ceramic materials with different Fe_2_O_3_ contents after sintering at (**a**) 900 °C, (**b**) 950 °C, (**c**) 1000 °C, and (**d**) 1050 °C.

Compressive strength was conducted on the samples of bauxite-tailing-based ceramic materials treated at different sintering temperatures with different Fe_2_O_3_ contents, and the compressive strength is shown in Figure 8. In Figure 8a, it can be concluded that at a sintering temperature of 900 °C, the compressive strength of ceramics is relatively low, and all values were lower than 60 MPa, indicating a low degree of ceramic sintering. When the sintering temperature is 950 °C, as shown in Figure 8b, with an increase in Fe_2_O_3_ content, the compressive strength of the ceramic first increases and then decreases. When the Fe_2_O_3_ content is 20 wt%, compressive strength reaches its maximum value of 91.89 MPa. At 950 °C, with the addition of Fe_2_O_3_, the initial increase in compressive strength with Fe_2_O_3_ addition is attributed to the fluxing effect of hematite, which promoted the vitrification of the clay mix. When the dosage of Fe_2_O_3_ is higher than 20 wt%, the decreasing amount of clay mineral resulted in a less amorphous phase, so particles in the ceramic body have not been well connected. At a sintering temperature of 1000 °C, the bauxite-tailing-based ceramics reach the optimal sintering temperature, and the compressive strength of ceramics is significantly higher than other temperatures. At this temperature, with the increase in Fe_2_O_3_ content, the compressive strength of ceramics first increases and then decreases. When the Fe_2_O_3_ content is 15 wt%, the compressive strength reaches its maximum, with an average of 120.81 MPa, which is better than 103.2 MPa for selling products. As shown in Figure 8d, when the Fe_2_O_3_ content is 15%, the compressive strength of 20 wt% ceramics is lower than 50 MPa at a sintering temperature of 1050 °C, resulting in lower strength and increased brittleness, which are caused by the excessive glass phase in the material. When the Fe_2_O_3_ content is 30 wt%, the reduction in the glass phase leads to an increase in the compressive strength of the material, but excessive Fe_2_O_3_ content further reduces the compressive strength of the ceramic. In summary, we conclude that the bauxite-tailing-based ceramic materials containing Fe_2_O_3_ can be prepared under the conditions of 950–1050 °C. When the highest Fe_2_O_3_ content is 30 wt%, the high bulk density and compressive strength of the ceramics can meet industrial needs. The recent industrial conditions are listed as follows: Fe_2_O_3_ content is required at 15 wt%, and the sintering temperature needs to be held at 1000 °C for 2 h.

**Figure 9:**
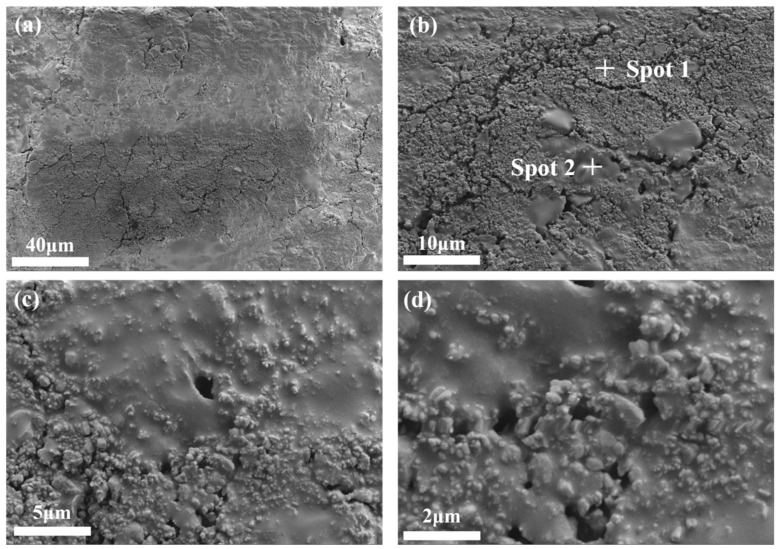
SEM images of the surface microstructure of bauxite-tailing-based ceramics sintered at 1000 °C with Fe_2_O_3_ contents at 15 wt%: (**a**) 500, (**b**) 2000, (**c**) 5000, and (**d**) 10,000.

SEM was conducted on bauxite-tailing-based ceramics with Fe_2_O_3_ contents of 15 wt% and a sintering temperature of 1000 °C. The microstructure images are shown in Figure 9, which shows the photos of the same area scanned at different magnifications. In Figure 9a, ceramics have a high densification under this preparation process. However, there is also a partial unevenness of the ceramic due to the changes in the composition of bauxite tailings. In Figure 9b–d, many glass phases are coated with grain particles, indicating that during the high-temperature sintering process, many liquid phases are generated, thereby promoting the densification of ceramics. The higher density results in smaller fluctuations in the volume density, apparent porosity, water absorption, and linear shrinkage of ceramics, while the uneven distribution of materials results in relatively large fluctuations with respect to compressive strength.

EDS analysis was performed in areas exhibiting different micro morphologies in Figure 9b, and their elemental weight percentages are shown in Table 3. The morphology of spot 1 is granular and condensed, and the morphology of spot 2 is paste. In the elemental composition of spot 1, the main elements are O, Al, and Si. Spot 2 shows the same elemental composition, but the Al element is significantly lower than that observed in spot 1. Based on the granular morphology of spot 1 and the amorphous morphology of spot 2, it is inferred that the granular material is composed of corundum and mullite, while the amorphous material comprises quartz glass.

**Figure 10:**
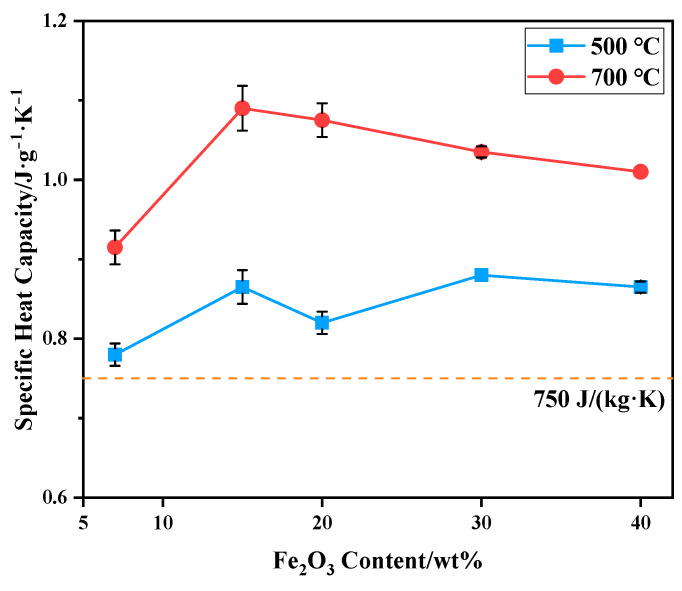
Specific heat capacity of bauxite-tailing-based ceramic materials with different contents of Fe_2_O_3_ at 500 °C and 700 °C.

The specific heat capacity of bauxite-tailing-based ceramic materials sintered at 1000 °C was tested at 500 °C and 700 °C, and the test results are shown in Figure 10. It can be observed in the figure that at a test temperature of 700 °C, the specific heat capacity of ceramics is higher than that at 500 °C, indicating that at a service temperature of 700 °C, the ceramic materials can better exert their heat storage capacity. With an increase in Fe_2_O_3_ content, the specific heat capacity of ceramics fluctuates at 500 °C. The specific heat capacity of ceramics at 500 °C and 700 °C is higher than 750 J/(kg·K), which meets industry-standard (JC-T 2135-2012) [32]. The specific heat capacity of the samples at 700 °C is higher than that of the product for sale, which is 940 J/(kg·K). At this temperature, with an increase in Fe_2_O_3_ content, the specific heat capacity first increases and then decreases, reaching a maximum of 1.06 J/(g·K) at a 15 wt% content of Fe_2_O_3_. Therefore, when 15 wt% Fe_2_O_3_ powder is added to bauxite-tailing-based ceramic materials and the sintering temperature is 1000 °C, as-prepared ferric-rich bauxite-tailing-based ceramic materials can be applied to RTO due to their high bulk density, low apparent porosity and water absorption, and high specific heat capacity.

## 4. Conclusions

(1) The phase transformation of bauxite tailings in the heating process is mainly from diaspore, kaolinite, and illite to mullite and corundum, and the products are finally mullite, corundum, quartz, and hematite. The high heat capacity properties of mullite and corundum and the high specific gravity of hematite can be used for the preparation of thermal storage ceramics
(2) In the preparation of bauxite-tailing-based ceramics, appropriate Fe_2_O_3_ can promote the formation of liquid phase, thus promoting the ceramic compact and phase transition of mullite, and corundum. A too-high Fe_2_O_3_ concentration will lead to the reduction of liquid phase components, which is not conducive to the preparation of ceramics.
(3) When the Fe_2_O_3_ content is 15 wt% and sintering temperature is 1000 °C, ferric-rich bauxite-tailing-based ceramic materials can be obtained with a bulk density of 2.53 g/cm^3^, apparent porosity of 9.64%, water absorption of 3.81%, linear shrinkage of 9.92%, compressive strength of 120.81 MPa and specific heat capacity of 1.06 J/(g·K), which meet the requirements of the industry and market. The preparation process plays an active role in the utilization of bauxite tailings and the reasonable development of inferior component Fe_2_O_3_.

## Figures and Tables

**Table 1:** Composition of bauxite tailings, lepidolite, and Fe_2_O_3_ powder.

Composition		Al_2_O_3_	SiO_2_	Fe_2_O_3_	K_2_O	TiO_2_	CaO	MgO	Li_2_O	Na_2_O	Others
Weight Percentage /wt%	Bauxite tailings	44.58	35.23	10.45	4.11	2.80	0.62	0.38	-	0.38	1.45
	Lepidolite	20.36	65.47	0.09	3.71	-	3.14	0.19	2.11	2.39	2.54
	Fe_2_O_3_ powder	-	-	99.99	-	-	-	-	-	-	-

**Table 2:** Designed raw materials and main chemical composition of samples.

Samples	Bauxite Tailing/g	Lepidolite/g	Fe_2_O_3_ Powder/g	Al_2_O_3_ Content of Mixtures/wt%	SiO_2_ Content of Mixtures/wt%	Fe_2_O_3_ Content of Mixtures/wt%
F7	67	33	0	35.59	45.21	7.03
F15	70	30	9.01	34.23	40.64	15.00
F20	70	30	15.82	32.33	38.25	20.00
F30	70	30	32.37	28.19	33.47	30.00
F40	42	18	32.66	24.16	28.69	40.00

**Table 3:** EDS element mass of the ceramic surface after sintering at 1000 °C with 15 wt% Fe_2_O_3_ content.

Element	O/wt%	Al/wt%	Si/wt%
Spot 1	77.09	13.87	9.04
Spot 2	87.07	4.98	7.95

## Data Availability

Not applicable.
